# The effects of a DTNBP1 gene variant on attention networks: an fMRI study

**DOI:** 10.1186/1744-9081-6-54

**Published:** 2010-09-16

**Authors:** Markus Thimm, Axel Krug, Thilo Kellermann, Valentin Markov, Sören Krach, Andreas Jansen, Klaus Zerres, Thomas Eggermann, Tony Stöcker, N Jon Shah, Markus M Nöthen, Marcella Rietschel, Tilo Kircher

**Affiliations:** 1 Department of Psychiatry and Psychotherapy, RWTH Aachen University, Pauwelsstr. 30, 52074 Aachen, Germany; 2 Department of Psychiatry and Psychotherapy, Philipps-University Marburg, Rudolf-Bultmann-Str. 8, 35039 Marburg, Germany; 3 Section of Brain Imaging, Philipps-University Marburg, Rudolf-Bultmann-Str. 8, 35039 Marburg, Germany; 4 Department of Neurology, Philipps-University Marburg, Rudolf-Bultmann-Str. 8, 35039 Marburg, Germany; 5 Institute of Human Genetics, RWTH Aachen University, Pauwelsstr. 30, 52074 Aachen, Germany; 6 Institute of Neuroscience and Medicine - 4, Forschungszentrum Jülich, 52425 Jülich, Germany; 7 Institute of Human Genetics, University of Bonn, Wilhelmstr. 31, 53111 Bonn, Germany; 8 Department of Genomics, Life & Brain Center, University of Bonn, Sigmund-Freud-Strasse 25, 53127 Bonn, Germany; 9 Department of Genetic Epidemiology in Psychiatry, Central Institute of Mental Health, J 5, 68159 Mannheim, Germany

## Abstract

**Background:**

Attention deficits belong to the main cognitive symptoms of schizophrenia and come along with altered neural activity in previously described cerebral networks. Given the high heritability of schizophrenia the question arises if impaired function of these networks is modulated by susceptibility genes and detectable in healthy risk allele carriers.

**Methods:**

The present event-related fMRI study investigated the effect of the single nucleotide polymorphism (SNP) rs1018381 of the *DTNBP1 *(dystrobrevin-binding protein 1) gene on brain activity in 80 subjects while performing the attention network test (ANT). In this reaction time task three domains of attention are probed simultaneously: alerting, orienting and executive control of attention.

**Results:**

Risk allele carriers showed impaired performance in the executive control condition associated with reduced neural activity in the left superior frontal gyrus [Brodmann area (BA) 9]. Risk allele carriers did not show alterations in the alerting and orienting networks.

**Conclusions:**

BA 9 is a key region of schizophrenia pathology and belongs to a network that has been shown previously to be involved in impaired executive control mechanisms in schizophrenia. Our results identified the impact of *DTNBP1 *on the development of a specific attention deficit via modulation of a left prefrontal network.

## Background

Attention deficits belong to the most prominent impairments among a wide range of cognitive deficits in schizophrenia patients [1]. In recent theories the concept of attention has been divided into three subdomains including "alerting", "orienting" and "executive control" [2,3]. Alerting comprises the cognitive control of wakefulness and arousal (intrinsic alertness) and the ability to increase response readiness for a short period of time subsequent to external cues (phasic alertness) [4]. Orienting refers to the overt or covert directing of spatial attention to unattended stimuli. Executive control of attention requires the ability to respond to one aspect of a stimulus while ignoring another (more dominant) aspect. In schizophrenia, deficits have been demonstrated in each of these three attentional subdomains [5-8]. To a lesser extent, impaired attention can also be found in non-psychotic relatives of schizophrenia patients. Accordingly, attention deficits might index genetic liability [9-13].

Investigating the neural correlates of attention deficits in schizophrenia patients and groups at genetic risk compared to healthy subjects might elucidate the mechanisms how genes can lead to the manifestation of a cognitive deficit. In healthy subjects, functional imaging studies have shown distinct neural networks underlying each of the three above mentioned attentional domains. Alerting has been shown to rely on thalamic, prefrontal and parietal areas [4,14-16]. Orienting has been associated with activation of the superior parietal lobe, temporal parietal junction, and frontal eye fields [14,17-19]. Executive control of attention has been consistently related to activation of the anterior cingulate and lateral prefrontal cortex [14,20-23]. While networks of alerting and orienting are lateralized to the right hemisphere [3,4,18], executive control shows a left lateral prefrontal bias [23,24].

Functional imaging studies have revealed dysfunctions of these networks in schizophrenia patients associated with the performance of attentional tasks requiring alerting, orienting or executive control [25-27]. Likewise, in relatives of schizophrenia patients, alterations have been found in networks of various cognitive domains [28] and in particular of the attention systems [29-31]. Neural abnormalities in relatives were similar to those in schizophrenia patients, and it was suggested that reduced BOLD activity in attention networks may be an intermediate marker for schizophrenia [31].

The studies in relatives have shown that genetic liability *in general *affects neural correlates of attention. However, schizophrenia has a polygenetic pattern of heredity and the impact of single genes on neural networks of cognition is largely unknown. Fan et al. [32] found that polymorphisms in dopamine receptor (*DRD4*) and monoamine oxidase A (*MAOA*) genes led to impaired executive control of attention in healthy subjects and that this was associated with reduced neural activity of the anterior cingulate cortex. The effect of (other) single gene variants on neural networks in healthy subjects has recently also been shown for other cognitive domains [33-41].

Several susceptibility genes for schizophrenia have been detected in recent years, including *DTNBP1*, neuregulin 1 (*NRG1*), catechol-O-methyltransferase (*COMT*), disrupted-in-schizophrenia 1 (*DISC1*), regulator of G-protein signalling 4 (*RGS4*), *G72*, proline dehydrogenase (*PRODH*), and D-amino acid oxidase (*DAAO*) [42-44]. Among these, *DTNBP1 *stands out as one of the best replicated susceptibility genes [45,46]. It has been shown to affect personality traits [47,48], intelligence [49-53], attention capacity [47], verbal fluency [37,52] and several memory domains [52-55] in both healthy subjects and patients with schizophrenia. In particular, negative symptoms in schizophrenia have been shown to be associated with several SNP of the *DTNBP1 *gene [56,57].

The effect of *DTNBP1 *on cognitive functions has been supposed to be mediated by the glutamate neurotransmitter system, acting via the prefrontal cortex [58]. In a gene expression study, Weickert et al. [59] showed that *DTNBP1 *mRNA levels varied depending on the *DTNBP1 *genotype and that schizophrenia patients showed significantly reduced *DTNBP1 *mRNA levels in the dorsolateral prefrontal cortex. Several SNPs of *DTNBP1 *have been detected and discussed as risk factors for schizophrenia [60]. In particular, the minor T allele of the SNP rs1018381 (P1578) which has a frequency of about 9% in the Caucasian population [61] can be regarded as an important risk factor. It has been shown to be strongly associated with schizophrenia in two of three independent samples of different ethnic origin (white and Hispanic, but not African American; [62]. In a study by Burdick et al. [49], this SNP was also the only one that showed a significant effect on general cognitive ability in patients and controls. Finally, a recent study on healthy individuals by Luciano et al. [52] provided further evidence for an association between the rs1018381 minor T allele and cognitive deficits in an Australian and Scottish cohort and a trend for an association was found for the English cohort.

The aim of the present study was to investigate the effects of SNP rs1018381 of the *DTNBP1 *gene on neural attention networks of alerting, orienting and executive control in healthy subjects. As an activation paradigm we used a modified version of the attention network test (ANT) developed by Fan et al. [63]. This test is capable of probing the three attention networks in one single reaction time task. Gene effects were expected to occur in brain areas known to be involved in alerting, orienting or executive control and to be impaired in schizophrenia patients. We hypothesized that risk allele carriers show reduced neural activity in

(1) prefrontal and parietal areas (with a right hemisphere bias) associated with alerting.

(2) superior parietal areas and the temporal parietal junction (with a right hemisphere bias) associated with orienting.

(3) anterior cingulate cortex and lateral prefrontal areas (with a left hemisphere bias) associated with executive control of attention.

## Materials and methods

### Subjects

Eighty participants were recruited from the RWTH Aachen university. Inclusion criteria were age (18-55 years), right-handedness (assessed by the Edinburgh Laterality Scale [64]) and no psychiatric disorder according to ICD-10. The study protocol was approved by the local ethics committee and each participant gave written informed consent.

### Genetic analysis

DNA was obtained from peripheral lymphocytes by a simple salting out procedure. The SNP rs1018381 [60] was genotyped using Applied Biosystems 7900HT Fast Real-Time PCR System and TaqMan-probes designed by Applied Biosystems (Foster City, California). For detection of the SNP rs1018381 the following Primers and VIC/FAM-probe sequences were used: Forward-5'- GAGTTACAAGTAAATGAAACGTCATGCA-3'; Reverse-5'-GCTGAGATCTGCCGGTGATTC-3'; 5'-VIC-ACAGC**G**TGCGGAAC-3'; 5'-FAM- AACAGC**A**TGCGGAAC. Note, that compared to previous studies by other groups, the common C allele is equivalent to our G allele and analogous the risk T allele [49,60,62] is equivalent to our A allele. The allele distribution in our total sample did not differ from the expected frequencies of SNP rs1018381 genotypes in a Caucasian sample [61]: 0,008 (A/A), 0,162 (A/G) and 0,830 (G/G). This was verified by Hardy-Weinberg equilibrium (HWE) using Haldane's exact test [65].

### fMRI task and stimuli

**Figure 1:**
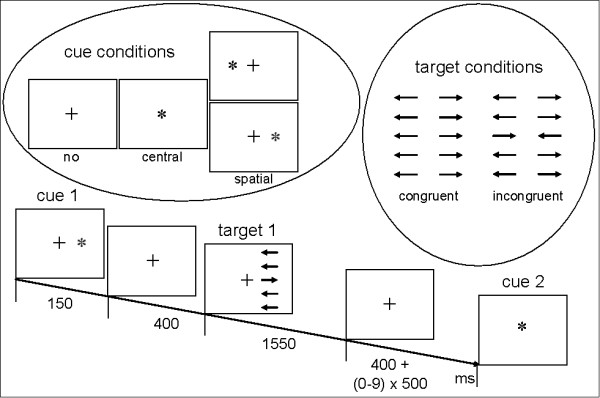
Stimulus material and example for the course of one trial (right spatial cue/incongruent target)

The fMRI task was designed with "Presentation" software (Neurobehavioral Systems Inc., San Francisco, CA) and was a modified version of the original attention network test (ANT) by Fan et al. [63]. Three cue conditions (no cue, center cue, spatial cue), two target conditions (congruent, incongruent) and two visual field conditions (left, right) were used (see Figure 1). As a cue an asterisk was used, appearing either at the center of the screen where it replaced a fixation cross (center cue) or lateralized next to the fixation cross, where it indicated the location of the following target stimulus (spatial cue). Targets consisted of a block of 5 parallel horizontal lines, with arrowheads pointing leftward or rightward. Target blocks were displaced approximately 4 degrees either left or right from the center (visual field condition). The middle of the 5 arrows was the target stimulus which had to be attended to. It was located at the horizontal midline. The other 4 arrows (two above and two below the target stimulus) served as distractors. In each trial, all distractor arrows pointed either to the same direction (congruent condition) or to the opposite direction (incrongruent condition) as the target arrow. Stimulus material was presented in black against a white background.

Each trial started with the presentation of a cue (or the fixation cross in the "no cue" condition) that was displayed for 150 ms. Following an interstimulus interval (ISI) of 400 ms (meanwhile the fixation cross was presented), the target (comprising target arrow and distractor arrows) appeared. Participants were required to indicate the direction of the target arrow by pressing a button with the left or right index finger corresponding to the left or right direction of the target arrow, respectively. Targets were presented for 1550 ms and followed by a presentation of the fixation cross until the end of the trial (i.e. presentation of the next cue). The duration of the fixation cross was 400 ms plus a pseudo-randomized multiple (0-9 times) of 500 ms, resulting in a total ISI of 400-4900 ms. Twenty trials were presented for each of the 12 possible permutations of conditions in a pseudo-randomized order, resulting in a total of 240 trials.

### Data acquisition

FMRI was performed on a 3-T Trio MR scanner (Siemens, Erlangen, Germany) in the Institute of Neuroscience and Medicine - 4, Research Centre Jülich, using a T2*-weighted echo planar imaging (EPI) sequence (time repetition = 2200 ms, time echo = 30 ms, flip angle = 90°). Slices covered the whole brain and were positioned transaxially parallel to the anterior-posterior commissural line (AC-PC). A total of 391 functional images were acquired, each consisting of 36 slices (3 mm thickness, 20 × 20 cm field of view, 64 × 64 image matrix). The initial three images were excluded from further analysis in order to remove the influence of T1 stabilization effects.

### fMRI data analysis

Analysis of fMRI data was done by SPM5 [66]. Functional images were realigned to the first image, normalized to the mean image (to a voxel size of 2 × 2 × 2 mm), smoothed (6 mm isotropic Gaussian filter) and high-pass filtered (cut off period 128 s). Temporal autocorrelations were removed using an autoregressive model of order 1 (AR(1)). Trials with incorrect or missing responses were removed. Twelve onset regressors (related to the onset of the targets) were defined resulting from the permutation of all conditions comprising 3 cues (no/center/spatial), 2 targets (congruent/incongruent) and 2 target positions (left/right). The hemodynamic response to each different event type was modeled using a canonical synthetic hemodynamic response function. The 6 head movement parameters were included as confounds. In order to identify the effects of a) alerting, b) orienting and c) executive control, first level linear contrasts were calculated, comparing a) events of center cue versus no cue b) events of spatial cue versus center cue and c) events of incongruent targets versus congruent targets. At the second level, the individual β-contrasts of the first level analyses were used to calculate t-tests to investigate for genotype effects (G/A vs. G/G) and for task effects in the whole sample regardless of risk/non-risk status. Effects were expected to occur in brain areas known to be involved in alerting, orienting or executive control and to be impaired in schizophrenia patients [25-27]. According to the present literature, specific regions of interests (ROIs) for each attentional subdomain were defined as follows: alerting = right hemisphere prefrontal and parietal areas [3,4,14-16]; orienting = right hemisphere parietal areas [3,14,17-19]; executive control of attention = left hemisphere prefrontal areas [14,20-24]. The prefrontal areas included the superior, middle and inferior frontal gyrus as well as the ACC. Analyses were performed with a predefined threshold of p < .001 (uncorrected). According to Thirion and coworkers [67] this is the optimal threshold in fMRI studies with regard to sensitivity and reliability. We are aware of the problem that the absence of correction for multiple testing enlarges the risk to commit the alpha error, i.e. to assume a between group difference that in reality does not exist. However, we consider that there are two reasons that legitimate our procedure: 1. We have a hypothesis about the areas that we expect to be activated and only look for results in predefined areas. 2. The disadvantage of a (conservative) correction for multiple comparisons is that the more you guard against the alpha error the higher the risk is to commit the beta error, i.e. *not *to detect an existing difference. The exploratory character of our results in the still relatively new research field of genetic imaging favours the use of this rather liberal significance threshold.

### Behavioral data analysis

Attentional effects were calculated as differential (d) scores from reaction times and error rates as follows: (1) alerting = (no cue) - (center cue); (2) orienting = (center cue) - (spatial cue); (3) executive control = (incongruent targets) - (congruent targets). Group differences (risk vs. non risk) for each attentional effect were proved by ANOVA.

## Results

### Subjects

Genetic analysis of SNP rs1018381 revealed 29 subjects with the risk allele variant A/G and 51 subjects with the non risk variant G/G. Risk group and non risk group did not differ concerning sex ratio, age, education and estimated IQ (see Table 1).

**Table 1:** Sociodemographic variables of the sample; means (standard deviations in parentheses)

Sample (n = 80)	Risk group (n = 29)	Non risk group (n = 51)	Statistics	p
Sex ratio (men/women)	22/7	32/19	χ^2 ^= 1.45	n.s.
Age	22.6 (2.3)	23.6 (3.2)	T = 1.57	n.s.
Education (years)	15.3 (2.2)	15.9 (2.8)	T = 0.94	n.s.
Estimated IQ	113.8 (11.3)	111.0 (13.2)	T = 0.93	n.s.

IQ was estimated using the MWT-B (Lehrl, 2005);

n.s. = non significant (p > .05);

### Behavioral data

**Figure 2:**
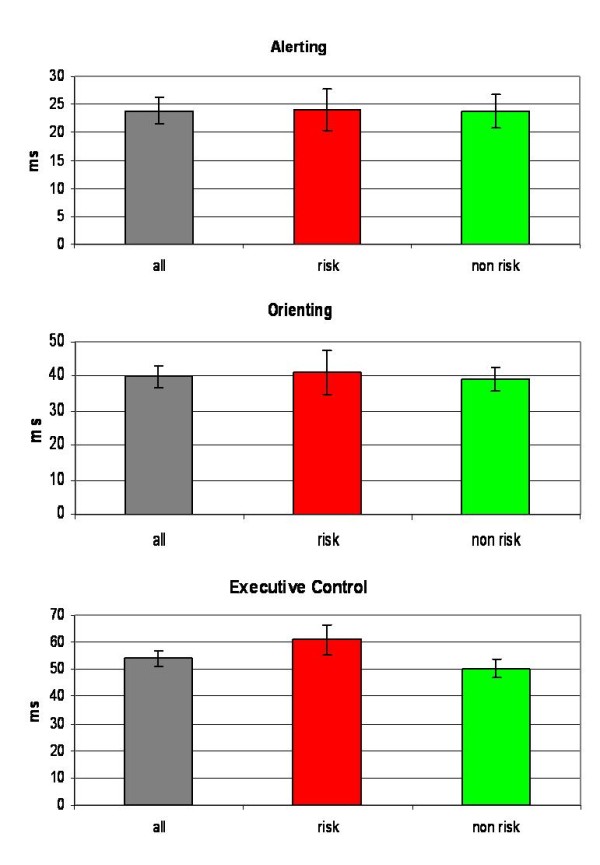
Effect of genotype on attentional effects

The total group showed significant effects in alerting (d = 24 ± 21 ms; T = 10.1; p < .001), orienting (d = 40 ± 28 ms; T = 12.9; p < .001) and executive control (d = 54 ± 26 ms; T = 18.7; p < .001). The executive control effect was significantly greater (T = 1.80; p = .038) in the risk group (d = 61 ± 29 ms) than in the non risk group (d = 50 ± 23 ms; see Figure 2). No group differences were found for the alerting and orienting effect.

### fMRI data

**Figure 3:**
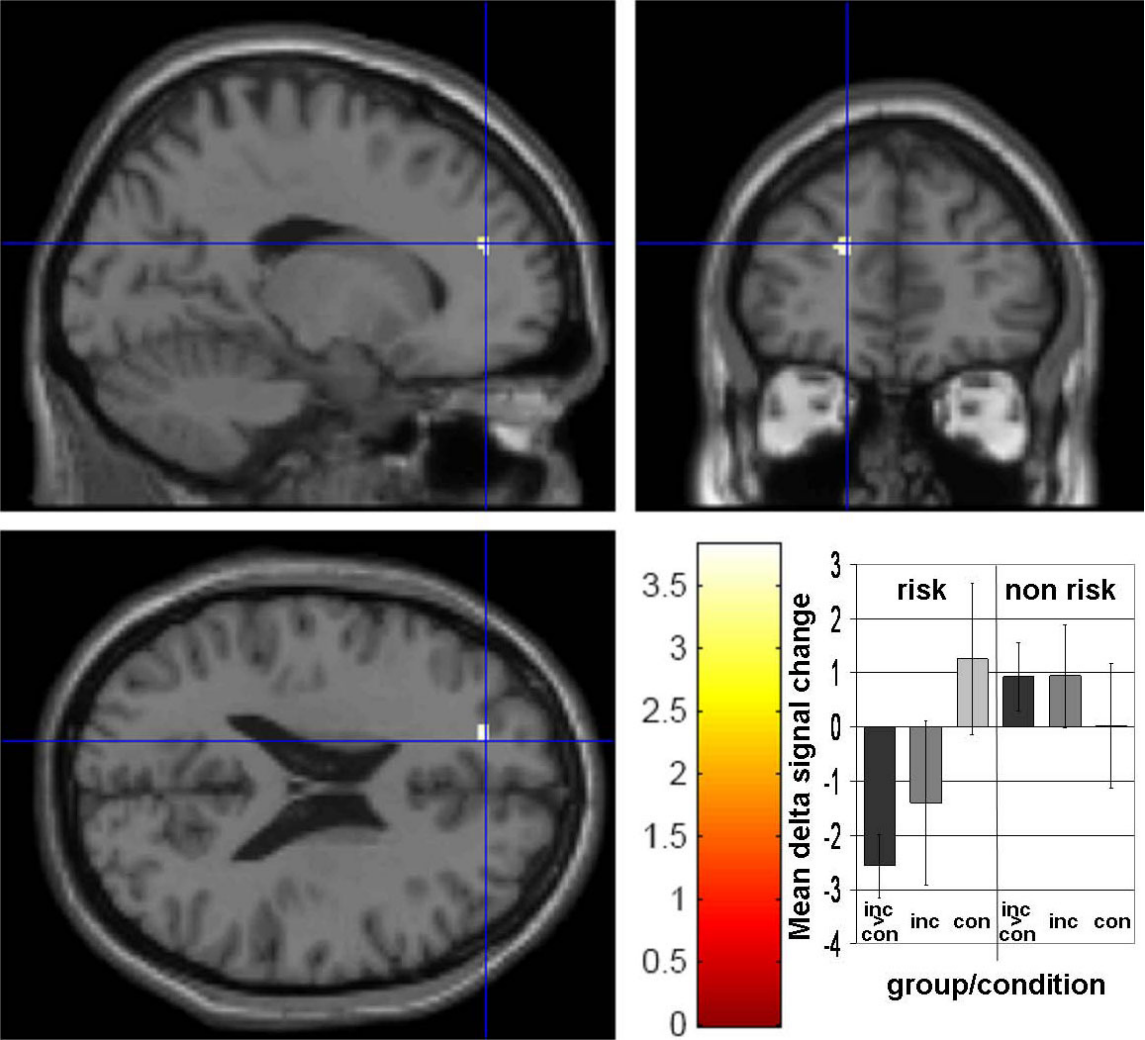
Decreased neural activity during executive control of attention risk group (inc > con) <non risk group (inc > con); bar graph shows mean betas of the GLM (arbitrary units)

Group differences were investigated for alerting, orienting and executive control in the predefined ROIs. During executive control, the group of risk allele carriers showed reduced neural activity in the left BA 9 of the superior frontal gyrus (Talairach coordinates [TC]: -18 44 18; cluster size [k]: 13 voxel; T = 3.81; p < .001 uncorrected, see Figure 3). A within group analysis of the total group (without respect to the genotype) revealed neural activity in a left prefrontal area. This was located in the white matter close to the middle/inferior frontal gyrus (TC: -32 39 2; k: 10 voxel; T = 3.41; p < .001 uncorrected). No group differences were found for alerting and orienting. A correlation between the degree of activation in the above mentioned cluster and the behavioral performance of executive control revealed a Pearson coefficient = -.099 which was not significant (p = .19).

## Discussion

The present fMRI study investigated the effect of the SNP rs1018381 of the *DTNBP1 *gene on attention networks. We used the well-established attention network test [14] to examine the three attentional domains "alerting", "orienting" and "executive control". No genotype effect was found for the alerting and orienting condition. However, in the executive control condition, risk allele carriers showed reduced neural activity in the left superior frontal gyrus (BA 9) when compared to the non risk control group. This was associated with a behavioral deficit in executive control that was reflected by a greater reaction time difference between the "congruent" and the "incongruent" condition where misleading information of distractors caused response conflict and had to be suppressed.

These results are well in line with previous studies showing that left lateral prefrontal cortex and ACC are the key regions of the executive control network in healthy subjects [14,20-24]. Furthermore these results are in accordance with schizophrenia pathology of cognition and neural functioning. In schizophrenia, deficits in executive control as well as dysfunction of the left prefrontal cortex and ACC are frequently reported findings [7,68-71]. A direct association between executive dysfunction and prefrontal cortex dysactivation in schizophrenia has been demonstrated in functional imaging studies [25,27]. Recent studies have shown that relatives of schizophrenia patients tend to have the same dysfunctions in an attenuated degree on the behavioral and neural level [28-31]. However, there is very limited evidence about the effects of single genetic variants on attention networks. In a behavioral study, Fossella et al. [72] found associations between polymorphisms of four candidate genes (*DRD4, DAT, COMT, MAOA*) and reduced efficiency specifically for executive attention. In a subsequent fMRI study, the effects of *DRD4 *and *MAOA *genes were shown to be associated with less activity in the ACC [32]. These dopaminergic genes had been chosen for that study because they were likely to affect the investigated networks since the prefrontal key areas - particularly for the executive control network - are known to be dopaminergic brain areas. However, schizophrenia is a polygenetic disorder and genes coding for other transmitter systems such as glutamate are also likely to modulate attention networks via the ACC or lateral prefrontal cortex. It has been found that dopamine modulates prefrontal activity by affecting the excitability of glutamatergic neurons [69] and that this interaction is disturbed in schizophrenia patients [73]. Several studies have investigated the effect of *DTNBP1 *in the development of schizophrenia. They showed that *DTNBP1 *is involved in the pre-synaptic protein expression and release of glutamate [74] and that schizophrenia patients have reduced *DTNBP1 *mRNA levels especially in the prefrontal cortex [59]. It has been supposed that particular *DTNBP1 *alleles increase the risk for schizophrenia and affect cognitive functions mediated by the glutamate neurotransmitter system directly affecting the development, maturation, and adult function of the prefrontal cortex [54,58,59].

Consistent with the interaction between dopaminergic and glutamatergic neurons in the prefrontal lobe we now found that the glutamatergic *DTNBP1 *gene led to the same effects of disturbed function of the executive control network in healthy risk gene carriers as Fan et al [32] showed for the dopaminergic genes *DRD4 *and *MAOA*. Our effects were specific for the left hemisphere network of executive control of attention and no alterations were found in the right hemisphere networks of alerting and orienting. This result might be explained by the different relevance of transmitter systems for distinct brain regions and cognitive functions. Alerting and orienting have been shown to rely on norepinephrine and cholinergic networks, respectively [75,76] and therefore these attentional domains were less likely to be affected by glutamatergic gene effects than executive control of attention. Furthermore, disturbances of alerting or orienting have been found to be less pronounced in schizophrenia than executive control [7,77] and for this reason it was also less likely to find dysfunctions of those networks in healthy risk allele carriers.

Two fMRI studies by Markov et al. [37,40] have investigated the effects of *DTNBP1 *(SNP rs 1018381) on working memory and verbal fluency in healthy subjects. They found neural networks of risk allele associated activation which did not match the cluster of our study. This is not surprising since the underlying networks of these cognitive domains differ from those of executive control of attention.

### Limitations

A limitation of our study is that we only studied the most promising risk allele variant (A/G of the SNP rs1018381) and it would be interesting to investigate the effects of other SNPs as well. Furthermore, a direct comparison of non psychotic risk gene carriers with a group of schizophrenia patients would be desirable and might lead to a better understanding of the aetiology of attention deficits in schizophrenia.

## Conclusions

Summarizing, our results elucidate the role of the *DTNBP1 *gene in the development of a specific dysfunction of the neural network underlying executive control of attention. Healthy risk allele carriers showed a behavioral deficit in this cognitive domain that was associated with reduced neural activity in the left superior frontal gyrus (BA 9). This area is known as a key region of schizophrenia pathology and belongs to a network that has been shown previously to be involved in impaired executive control mechanisms in schizophrenia. Thus, our results suggest that executive control deficits in schizophrenia might be codetermined by *DTNBP1 *allele status and be based on a modification of a left prefrontal network that is already emerging in healthy risk allele carriers.

## Competing interests

The authors declare that they have no competing interests.

## Authors' contributions

TKi, MMN and MR conceived the core of the study design. AK, TKe, VM, SK, AJ, TS and NJS performed the acquisition of the fMRI data. KZ, TE, MMN and MR performed the genetic analyses. MT, AK and TKe carried out the statistical analysis of the data. MT carried out the interpretation of the results and composed the manuscript. AK, AJ and TKi also revised the manuscript critically. All authors read and approved the final manuscript.
